# Two new species of *Prionomastix* Mayr (Hymenoptera: Encyrtidae) with a key to Indian species

**DOI:** 10.3897/BDJ.4.e7326

**Published:** 2016-03-08

**Authors:** Tirunagaru Krishnachaitanya, Sagadai Manickavasagam

**Affiliations:** ‡ Annamalai University, Department of Entomology, Chidambaram, Tamil Nadu, India

**Keywords:** Chalcidoidea, Encyrtinae, Encyrtini, Prionomasticina, Bihar, Tamil Nadu, key

## Abstract

**Background:**

Species of *Prionomastix* Mayr were not known from India when Manickavasagam and Rameshkumar 2011 and Rameshkumar and Poorani 2015 misidentified a new species as P. siccarius. Now it is corrected by explaining the characters as to why it is new and not P. siccarius along with another new species. As we have one another new species, P. orientialis, described by Rameshkumar and Poorani 2015 our two new species are compared with P. orientalis also.

**New information:**

Two new species of *Prionomastix* Mayr (Hymenoptera: Encyrtidae), one from Bihar state and the other from Tamil Nadu state, India, are described *viz.*, *P. tamilnadensis* **sp. nov** and *P. biharensis* **sp. nov**. and a key to all known Indian species is provided.

## Introduction

*Prionomastix* was erected by Mayr 1876 for *Encyrtus morio* Dalman and it belongs to the subfamily Encyrtinae, tribe Encyrtini, subtribe Prionomasticina. Where the hosts are known, members of this genus are primary, solitary parasitoids of membracid and cicadellid nymphs (Hemiptera: Membracidae and Cicadellidae) (Prinsloo 1983; Annecke 1962; Trjapitzin and Myartseva 2002 and Trjapitzin 2005). Out of the 27 species known globally, the insect hosts are recorded only for six, *viz.*, *P. capeneri* Annecke ex. *Leprechaunus cristatus* Capener; *P. montana* Prinsloo ex. *Dukeobelus simplex* (Walker); *P. siccarius* Annecke ex. *Gongroneura fasciata* (Buckton) (Trjapitzin 2005) and *P. estrellus* Noyes ex. *Ennya chrysura* (Fairmaire) (Noyes 2010) all from nymphs of membracidae *P. wonjeae* Mbonji ex. nymphs of *Coloborrhis coricina camerunensis* Mbondji (nymphs of Cicadellidae) (Trjapitzin 2005) and the sixth *P. morio* as a parasitoid of larvae of Apionidae (Coleoptera) (Thompson 1954) is almost certainly erroneous (Noyes 2010). Totally 27 species are known globally Noyes 2015. Manickavasagam and Rameshkumar 2011 first reported *Prionomastix siccarius* Annecke, which they reared from a membracid nymphs on *Prosopis* sp. However, it was a misidentification and is a new species that we are publishing now as *P. tamilnadensis* sp. nov. along with *P. biharensis* sp.nov.

## Materials and methods

All measurements are in millimeters and taken initially from micrometer divisions directly fitted in the eye piece of a Leica S8APO stereo zoom trinocular microscope at 80× for card mounted specimens or at 100× using a Leica DM750 phase contrast microscope for slide mounted parts and finally converted into millimeters. Images of card mounted specimens were captured using a Leica M205C stereo zoom trinocular microscope with a DMC2900 camera, and those of slide mounted parts using a DFC295 camera attached to a Leica DM750 phase contrast microscope.

The following abbreviations are used:

AOL = Minimum distance between a posterior ocellus and the anterior ocellus.

F1, F2, etc. = Funicle segments 1, 2, etc.

OCL = Minimum distance between a posterior ocellus and the occipital margin.

OOL = Minimum distance between a posterior ocellus and the corresponding eye margin.

POL = Minimum distance betweenposterior ocelli.

The following acronyms are used for the depositories:

EDAU = Entomology Department, Annamalai University, Chidambaram, India.

NBAIR = National Bureau of Agricultural Insect Resources (Formerly NBAII), Bangalore, India.

## Taxon treatments

### Prionomastix

Mayr

Prionomastix Mayr 1876: 725−726. Type species *Encyrtus morio* Dalman, by monotypy.
Liocarus Thomson 1876: 115,121−122. Type species *Encyrtus morio* Dalman, by monotypy. Objective synonym of *Prionomastix*.
Chestomorpha Ashmead 1900: 370. Type species *Chestomorpha biformis* Ashmead, by original designation and monotypy. Synonymy with *Prionomastix* by Trjapitzin and Gordh 1978 (1978: 365).
Aprionomastix Girault 1913: 68. Type species *Aprionomastix fasciatipennis* Girault, by original designation and monotypy. Synonymy with *Prionomastix* by Annecke 1962 (1962: 504).
Prionomastix

#### Diagnosis

Female body measuring from 1.6−3.8mm in length, pale orange to dark brown or black, never metallic; fore wing infuscate or hyaline; hind wing hyaline; body robust. Head with occipital margin sharp; ocelli forming an angle of about 85−120°, posterior ocelli never closer to each other than to eye margin; antennal scrobes short, hardly longer than torulus, naked and meeting dorsally, inverted U or V shaped; antennal torulus relatively high on head, separated from mouth margin by at least about its own length; malar suture absent; mandible wide, widening towards apex; maxillary palpus two, three or four segmented, first and second segments sometimes partially or completely fused, labial palpus two or four segmented; scape subcylindrical; funicle six segmented; clava three segmented or entire. Mesoscutum with notaular lines absent; scutellum slightly convex; mid basitarsus with a single line of ventral pegs; postmarginal vein at least as long as stigmal, almost always much longer; stigmal vein strongly curved, uncus absent; linea calva entire and almost always closed; mesopleuron clearly separated from gaster by propodeum which more or less widely touches hind coxa; propodeum with numerous setae behind spiracle, these often extending down side to base of hind coxa. Gaster with cercal plates situated near apex of gaster; hypopygium reaching to about two-thirds along gaster or to apex; ovipositor curved upwards; ovipositor about 0.5−1.2× as long as mid tibia, not exserted, but sometimes extending upwards past apex of syntergum; second valvifer without subapical setae; gonostylus fused to second valvifer, naked, occasionally membranous (Noyes 2010).

*Male*: Body measuring from 1.0−1.9 mm in length. Generally similar to female but frontovertex slightly wider; antennal toruli relatively a little higher on head; antenna with segments wider than in female, with very prominent linear sensilla on all flagellar segments; clava solid; fore wings mostly hyaline, rarely distinctly infuscate; phallobase varying from short and stout to elongate and slender, with a short, distal, median ventral process; paramere distinct and elongate, about 3−4× as long as wide, naked, digitus varying from shorter than paramere and straight to considerably longer and curved, up to 9× as long as wide, without apical hooks, but with up to 5 subapical setae; aedeagus variable, from slender to very wide with simple rounded to bilobed apex, varying from 0.25−0.9× as long as mid tibia (Noyes 2010).

#### Notes

*Prionomastix* can be differentiated from other encyrtid genera using the keys given by Noyes and Hayat (1984) for Indo-Pacific region & Hayat 2006 for India. For other useful taxonomic discussions on this genus one can refer to Hoffer (1957), Annecke (1962), Gordh and Trjapitzin (1981), Trjapitzin (1989) and Trjapitzin and Ruiz-Cancino (1998) and Noyes (2010).

### Prionomastix tamilnadensis

Manickavasagam & Krishnachaitanya sp. n.

urn:lsid:zoobank.org:act:C4FF38DA-FA31-43AC-8B97-70781DCDA253

#### Materials

- **Type status:** Holotype. **Occurrence:** recordedBy: Manickavasagam and A. Rameshkumar; individualCount: 1; sex: female; lifeStage: adult; **Taxon:** scientificName: Prionomastix tamilnadensis; **Location:** country: India; stateProvince: Tamil Nadu; locality: Coimbatore, near Maruthamalai; verbatimElevation: 314 m; decimalLatitude: 11.0280171; decimalLongitude: 76.9020389; georeferenceProtocol: label; **Identification:** identifiedBy: *Prionomastix siccarius* Annecke: Manickavasagam & Rameshkumar, 2011: 112, female, male. India (Tamil Nadu) record. Misidentification.Manickavasagam & Krishnachaitanya; dateIdentified: 2015; **Event:** eventID: (EDAU, Registration No. Enc/011/2015).; samplingProtocol: Host rearing; eventDate: 09/22/2009; **Record Level:** language: english; collectionCode: Insects; basisOfRecord: PreservedSpecimen
- **Type status:** Paratype. **Occurrence:** recordedBy: Manickavasagam and A. Rameshkumar; individualCount: 1; sex: male; lifeStage: adult; **Taxon:** scientificName: Prionomastix tamilnadensis; **Location:** country: India; stateProvince: Tamil Nadu; locality: Coimbatore, near Maruthamalai; verbatimElevation: 314 m; decimalLatitude: 11.0280171; decimalLongitude: 76.9020389; georeferenceProtocol: label; **Identification:** identifiedBy: Manickavasagam & Krishnachaitanya; dateIdentified: 2009; **Event:** eventID: (EDAU, Registration No. Enc/011/2015); samplingProtocol: Host rearing; eventDate: 09/22/2009; **Record Level:** language: english; collectionCode: Insects; basisOfRecord: PreservedSpecimen

#### Diagnosis

**Figure 1.**
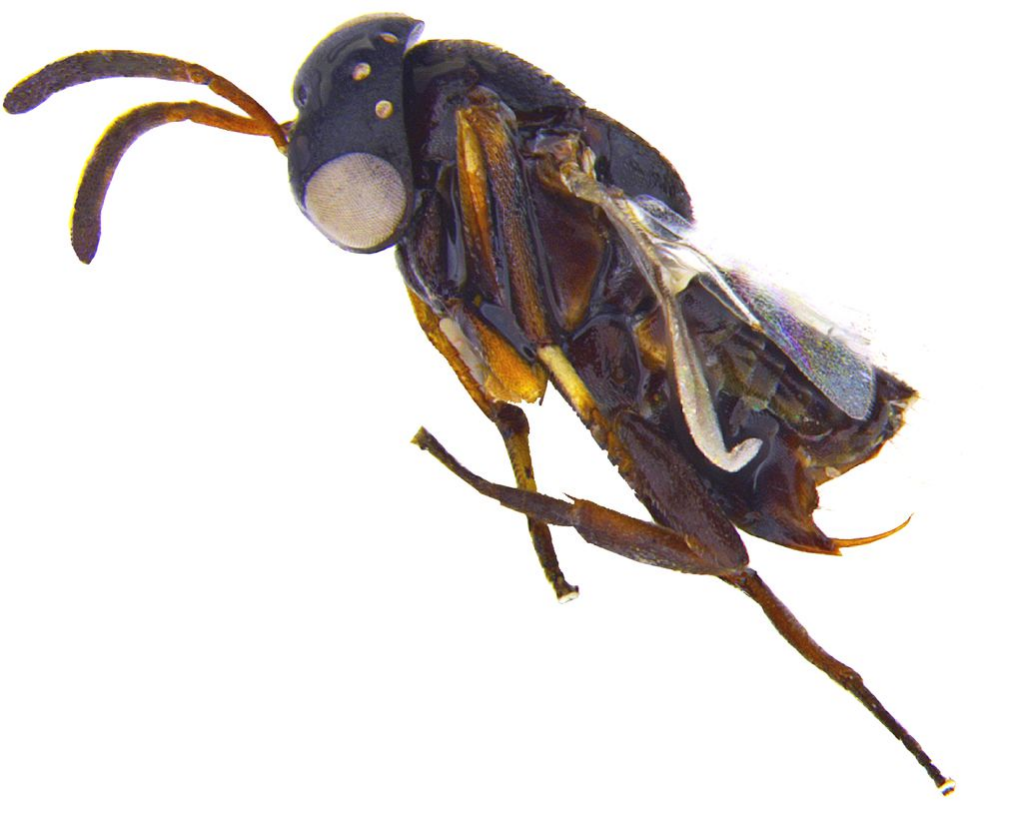
Habitus image Female

Body brown to dark brown with yellow patches (Fig. 1); Fore wing infuscate area around postmarginal and stigmalvein; maxillary palpus two segmented; ocelli forming an angle of about 110°; scape, 5.42× as long as wide; pedicel, 1.1× as long as wide; funicle with F1, 0.176× as long a wide and longer than F2−F6 individually; clava, three segmented, first segment long and as long as rest two segmented combined, apically more or less round; ovipositor 4.57× as long as mid tibial spur, curved and slightly exserted.

Female. Holotype (Fig. 1). Length, 2.75 mm. Head black except, torulus white; eye silvery white, ocelli white. Antenna with radicle testaceous; scape testaceous except, dorsoapically light brown; pedicel, funicle and clava dark brown. Thorax black with dark brown setae; tegula black, except apex yellowish. Fore wing infuscate area around postmarginal and stigmal vein. Mesopleuron anterially dark brown, fading out and posteriorly light brown; coxae brown; fore femur basal half brown, apical half yellow, tibia and tarsus yellow except apical tarsomere brown; mid femur and tibia brownish yellow, basitarsus white, except dorsal apex and rest of tarsi brown, mid tibial spur yellow with black apex; hind leg brown except, apical 1/3 of tibia paler. Propodeum dark brown; gaster dark brown except lateral base, apex of hypopygium and ovipositor yellowish brown.

**Figure 2.**
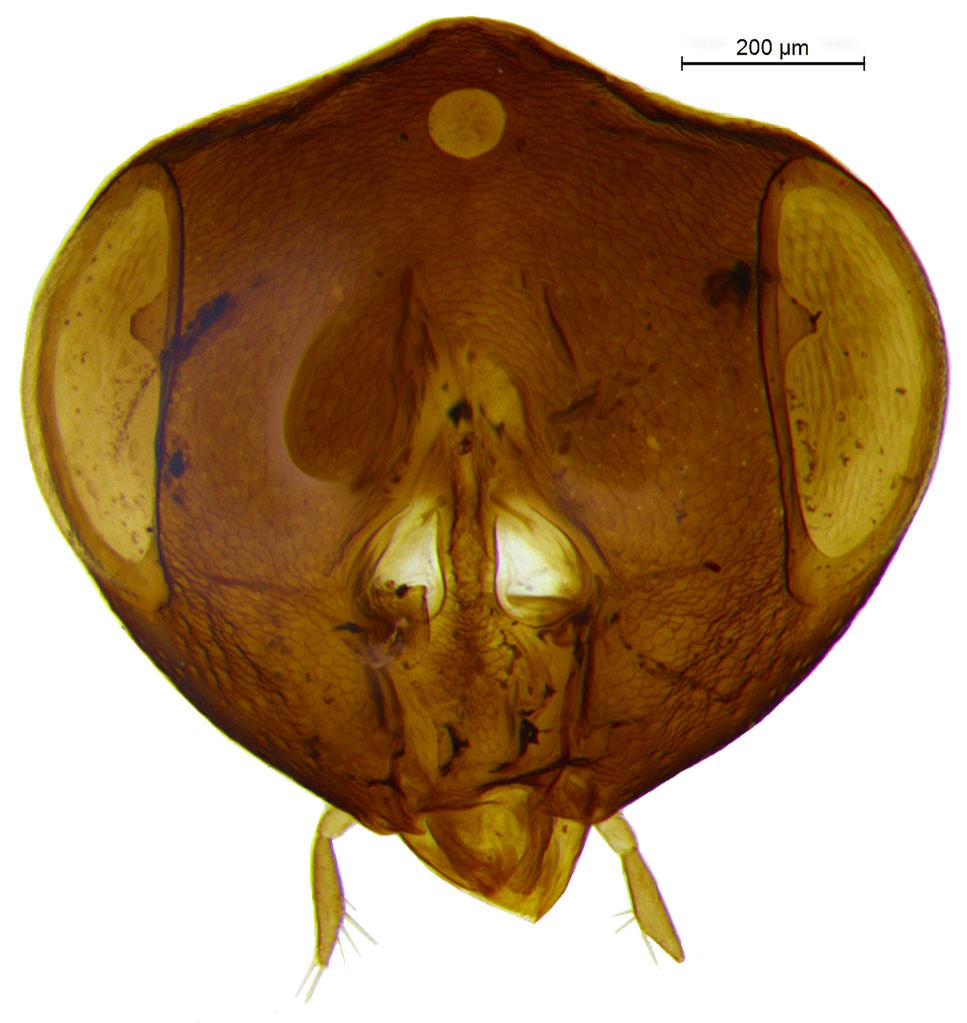
Head frontal view Female

**Figure 3.**
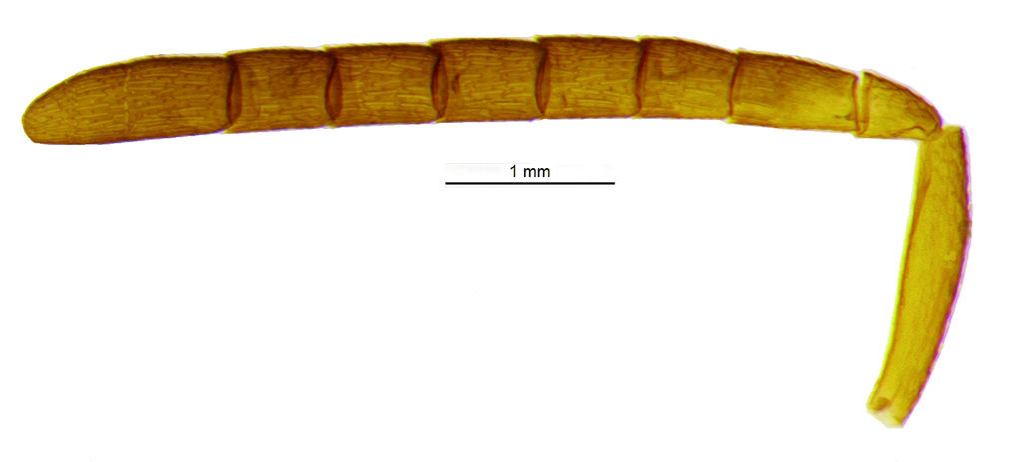
Antenna Female

Head (Fig. 2) in frontal view wider than high (1.02:0.88); frontovertex with imbricate sculpture, but cheek sharing coriarious-punctate; maxillary palp three segmented; ocelli forming an angle of about 110°; inter antennal prominence slightly raised; eye in frontal view 1.31× as long as wide. Antenna (Fig. 3) with scape slightly flattened, 5.42× as long as wide; pedicel, 1.1× as long as wide; funicle with F1, 1.76× as long a wide and longer than F2−F6 individually; clava, three segmented, first segment long and as long as rest two segments combined, apically more or less round; longitudinal sensillae present in all funicle segments, F1:F2:F3:F4:F5:F6, 19:28:41:47:59:51 and three claval segments with, 39:28:18 sensillae respectively. *Measurements*: (card mounted 80×) eye height:width, 0.5:0.38; OOL 0.075; OCL 0.031; POL 0.287; AOL 0.013. (slide mounted 100×) head height:width, 0.88:1.02; frontovertex width, 0.64; scape length:width, 0.38:0.07; pedicel length:width, 0.11:0.10; funicle length:width, F1, 0.15:0.085; F2, 0.11:0.10; F3, 0.12:0.105; F4, 0.12:0.105; F5, 0.13:0.10; F6, 0.12:0.10; clava length:width, 0.26:0.10.

**Figure 4.**
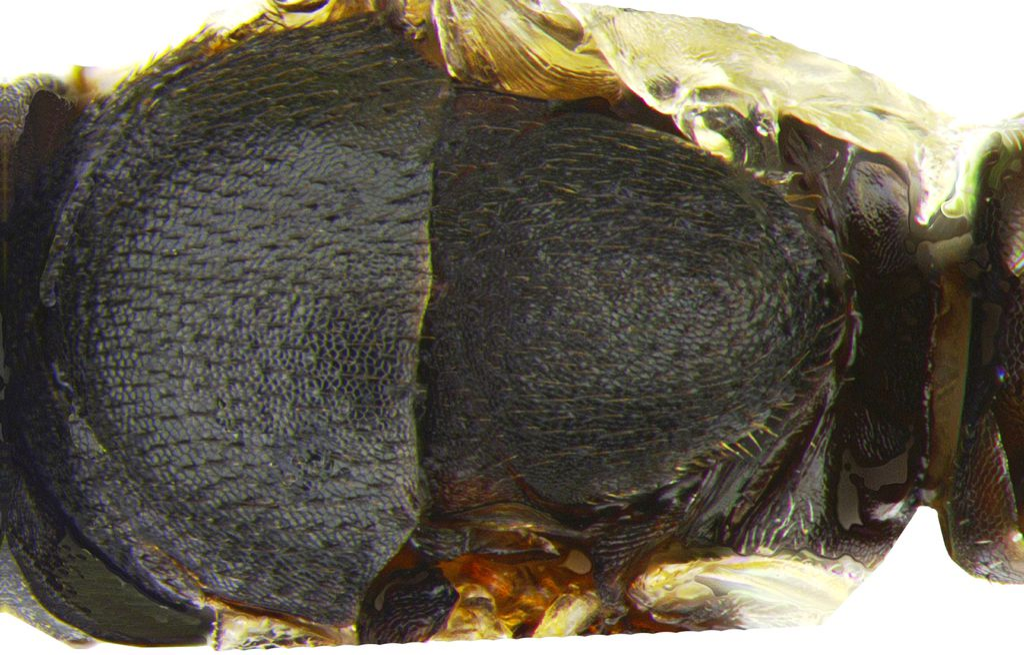
Dorsal view of nota Female

**Figure 5.**
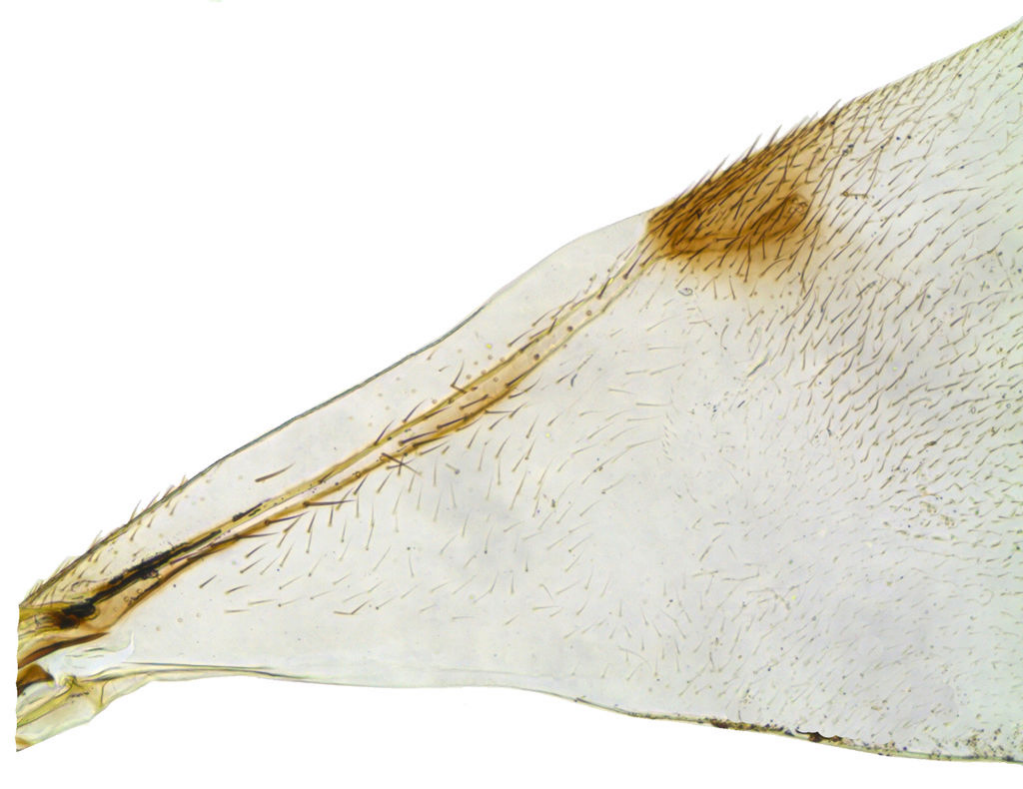
Fore wing Female

Mesosoma (Fig. 4) longer than gaster (1.31:1), with imbricate sculpture; mesoscutum 1.66× as wide as long; scutellum, 1.30× as wide as long. Fore wing (Fig. 5), 2.43× as long as wide; costal cell 9.77× as long as wide; postmarginal vein 1.5× as long as stigmal vein; hind wing, 2.33× as long as wide. *Measurements*: (card mounted 80×) mesosoma length:width, 1.31:0.63; mesoscutum length:width, 0.637:1.062; scutellum length:width, 0.575:0.75. (slide mounted 100×) fore wing length:width, 2.07:0.85, costal cell length:width, 0.88:0.09; postmarginal vein length, 0.24; stigmal vein length, 0.16; hind wing length:width, 1.4:0.6; hind tibia length:width, 0.825:0.187, hind basitarsus length, 0.35; mid basitarsus length, 0.375; mid tibial spur length, 0.237; mid tibia length 1.00.

**Figure 6.**
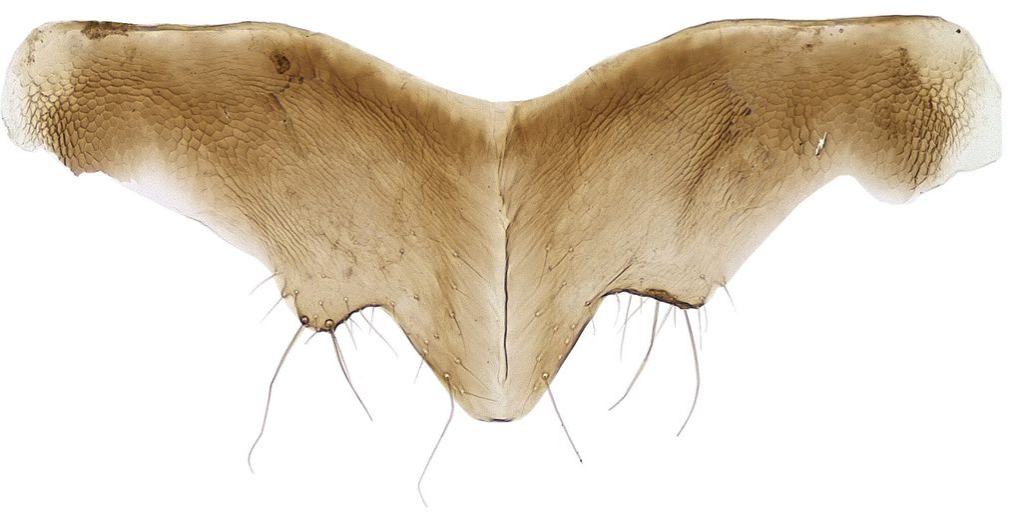
Hypopygium Female

**Figure 7.**
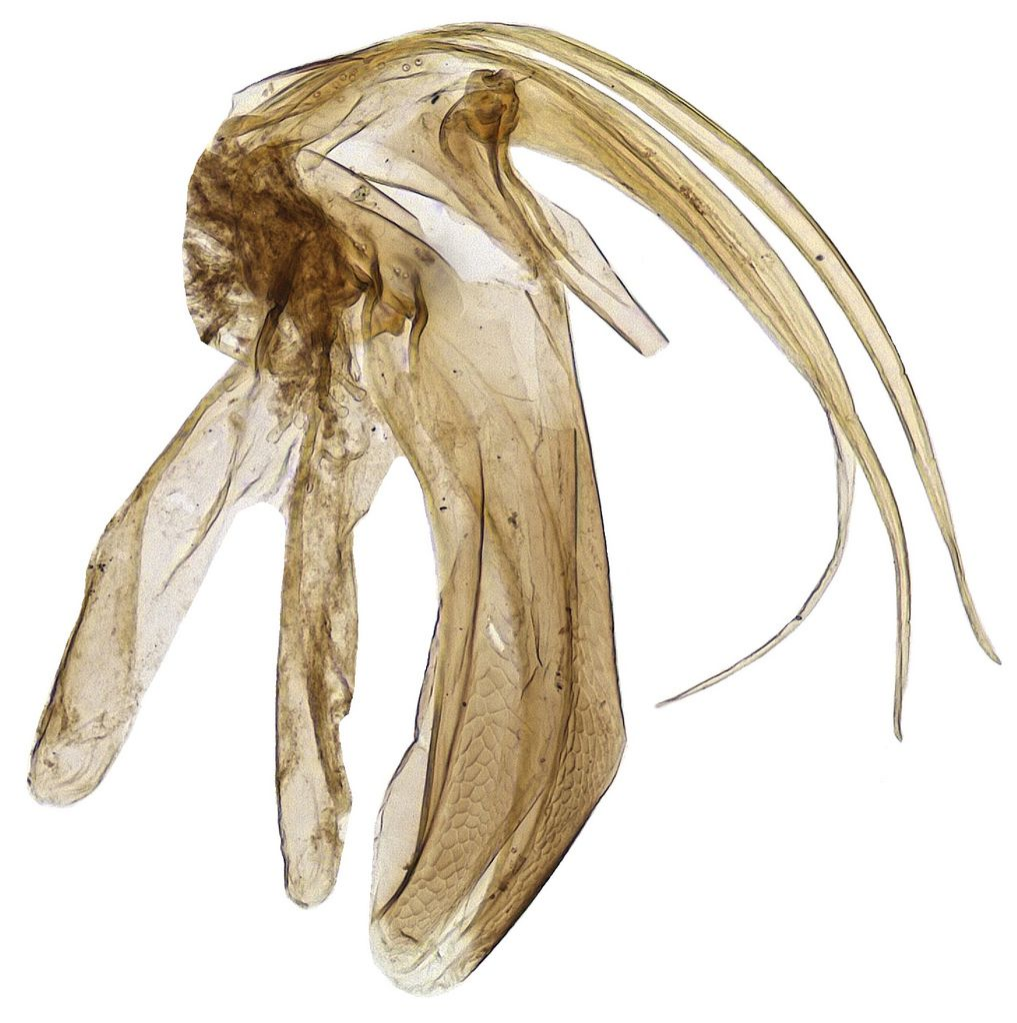
Ovipositor

Metasoma shorter than thorax; gaster imbricately sculptured; hypopygium (Fig. 6) nearly extending to apex of gaster; ovipositor (Fig. 7) 4.57× as long as mid tibial spur, curved and slightly exserted. *Measurements*: (card mounted 80×) metasoma length 1.00. (slide mounted 100×) ovipositor length 1.0875.

**Figure 8.**
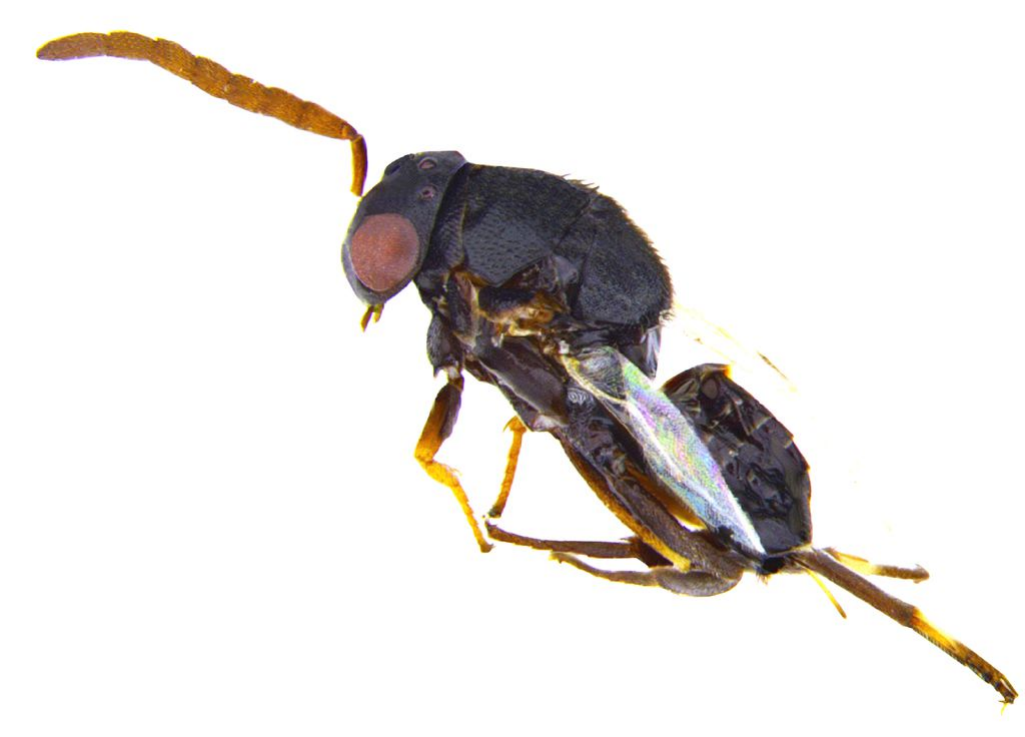
Habitus image Male

**Figure 9.**
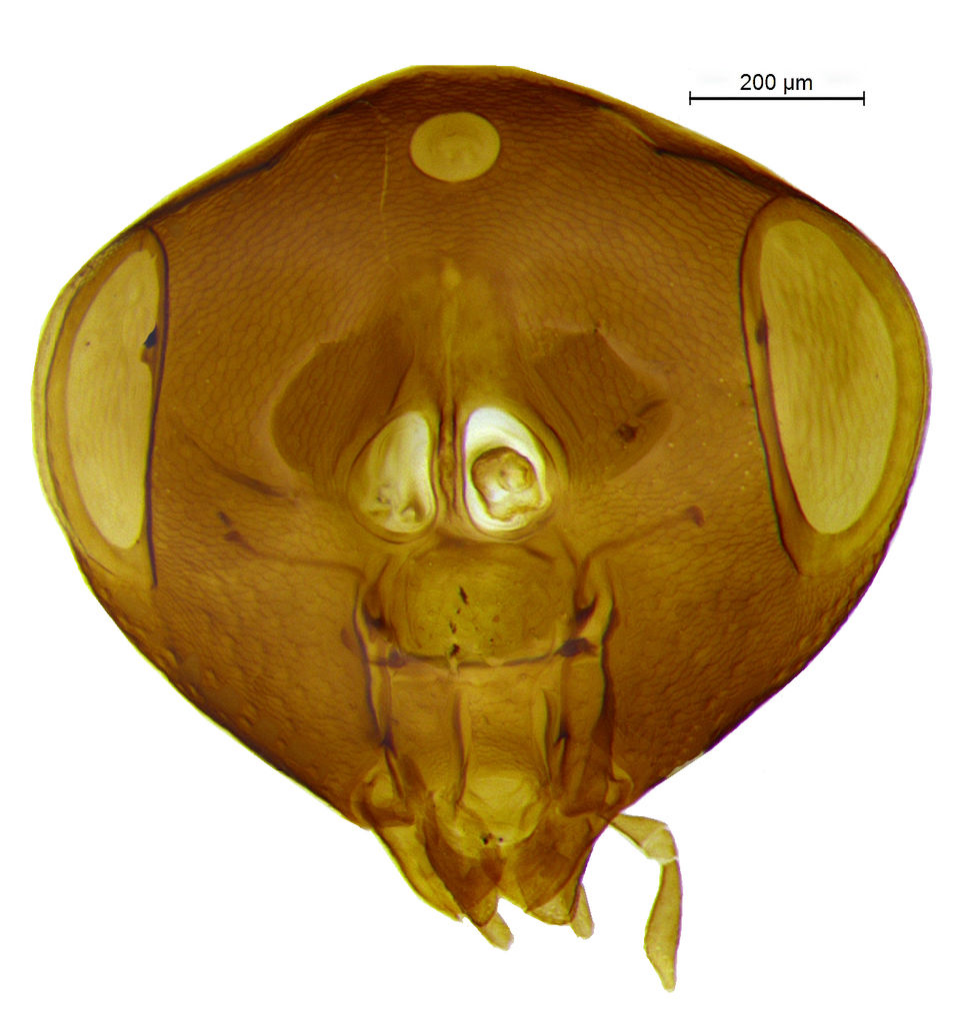
Head frontal view Male

**Figure 10.**
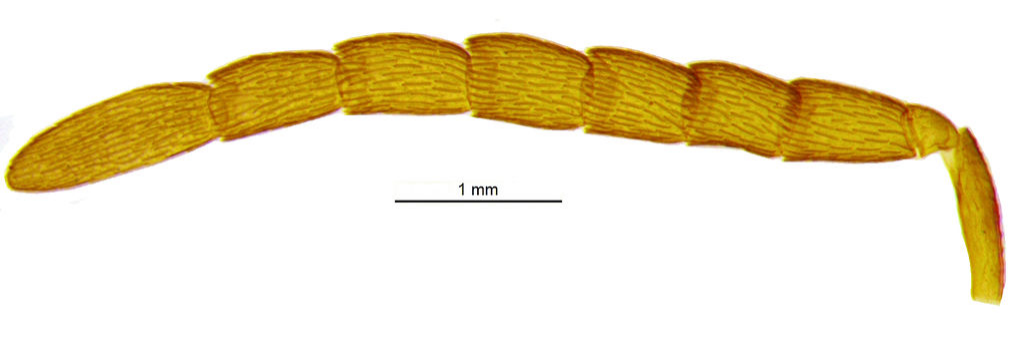
Antenna Male

**Figure 11.**
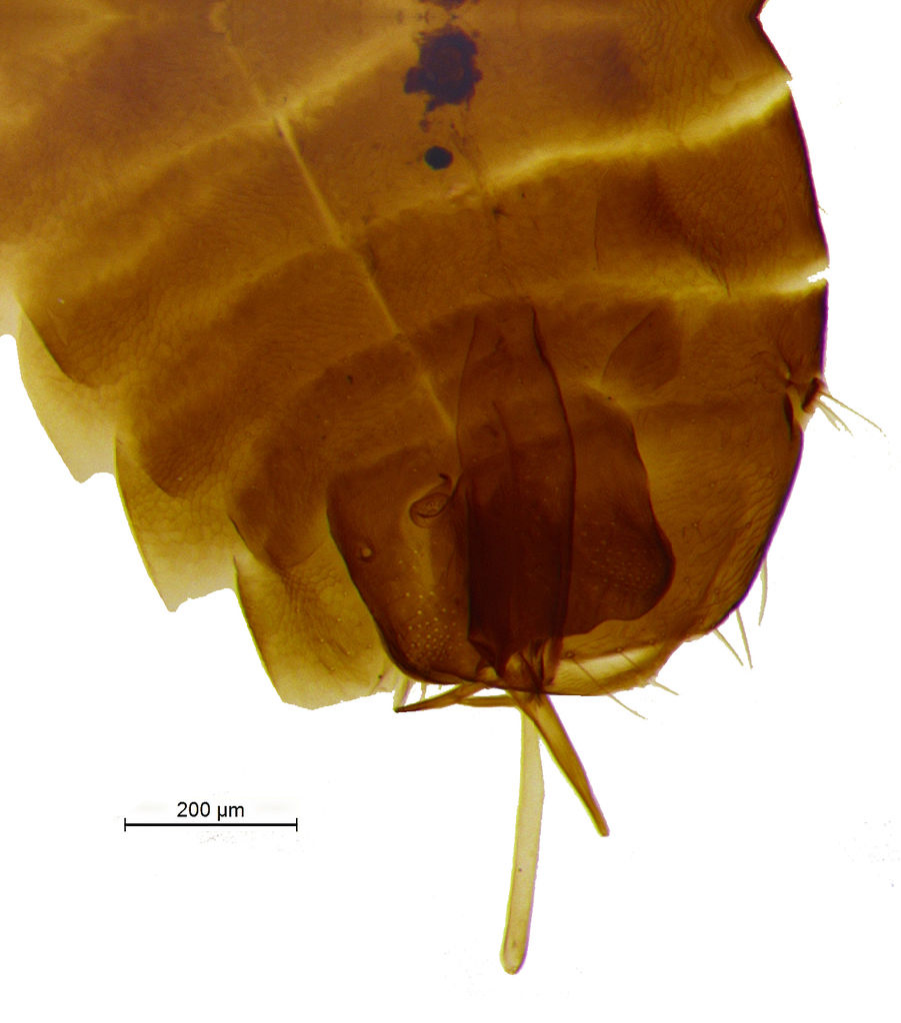
Male genitalia

*Male*: Similar to female (Fig. 8), except for maxillary palpi dimensions (Fig. 9) Antenna (Fig. 10), scape 4.76× as long as wide; funicle as in; clava entire. *Measurements*: (slide mounted 100×) ​scape length:width, 0.31:0.065; pedicel length:width, 0.1:0.08; funicle length:width, F1, 0.23:0.14; F2, 0.2:0.14; F3, 0.2:0.15; F4, 0.21:0.15; F5, 0.22:0.13; F6, 0.22:0.13; clava length:width, 0.4:0.13; fore wing length:width, 2.05:0.9, costal cell length:width, 0.95:0.09; postmarginal vein length, 0.28; stigmal vein length, 0.13; genitalia length 0.77 (Fig. 11).

**Host.** One pair ex. unidentified membracid nymphs on *Prosopis* sp.

#### Etymology

The species is named after the state from where it was collected.

#### Notes

This species is closer to *P. siccarius* in having the, mesosoma dorsally mostly black; F6 longer than wide and antenna dark brown, scape testaceous except, dorsoapically light brown, but differs in F1 1.36× as long as pedicel; fore wing with an infuscate area around postmarginal and stigma vein; fore femur basal half brown, apical half yellow, tibia and tarsus yellow except apical tarsomere brown (F1 nearly 2.0× as long as pedicel; fore wing with a single narrow subparallel transverse band from marginal to end of venation connecting anterior and posterior wing margins (fig. 8 of Annecke 1962); fore legs dark testaceous with faint blackish suffusions on the femora internally and tibiae externally in *P. siccarius*).

‘Prionomastix siccarius Annecke: Manickavasagam & Rameshkumar, 2011: 112, female, male. India (Tamil Nadu) record. Misidentification.’

### Prionomastix biharensis

Manickavasagam & Krishnachaitanya sp. n.

urn:lsid:zoobank.org:act:79F2311F-9FD6-48C9-A179-0D16692599C3

#### Materials

- **Type status:** Holotype. **Occurrence:** recordedBy: Abhinav Kumar; individualCount: 1; sex: female; lifeStage: adult; **Taxon:** scientificName: Prionomastix biharensis; **Location:** country: India; stateProvince: Bihar; locality: Banka, Bounsi; verbatimElevation: 79 m; decimalLatitude: 24.81065; decimalLongitude: 87.03857; georeferenceProtocol: label; **Identification:** identifiedBy: Manickavasagam & Krishnachaitanya; dateIdentified: 2014; **Event:** eventID: (EDAU, Registration No. Enc/012/2015); samplingProtocol: Yello Pan Trap; eventDate: 01/08/2014; **Record Level:** language: english; collectionCode: Insects; basisOfRecord: PreservedSpecimen
- **Type status:** Paratype. **Occurrence:** recordedBy: Abhinav Kumar; individualCount: 3; sex: female; lifeStage: adult; **Taxon:** scientificName: Prionomastix biharensis; **Location:** country: India; stateProvince: Bihar; locality: Banka, Bounsi; verbatimElevation: 79 m; decimalLatitude: 24.81065; decimalLongitude: 87.03857; georeferenceProtocol: label; **Identification:** identifiedBy: Manickavasagam & Krishnachaitanya; dateIdentified: 2014; **Event:** eventID: (EDAU, Registration No. Enc/012/2015); samplingProtocol: Yello Pan Trap; eventDate: 01/08/2014; **Record Level:** language: english; collectionCode: Insects; basisOfRecord: PreservedSpecimen

#### Diagnosis

**Figure 12.**
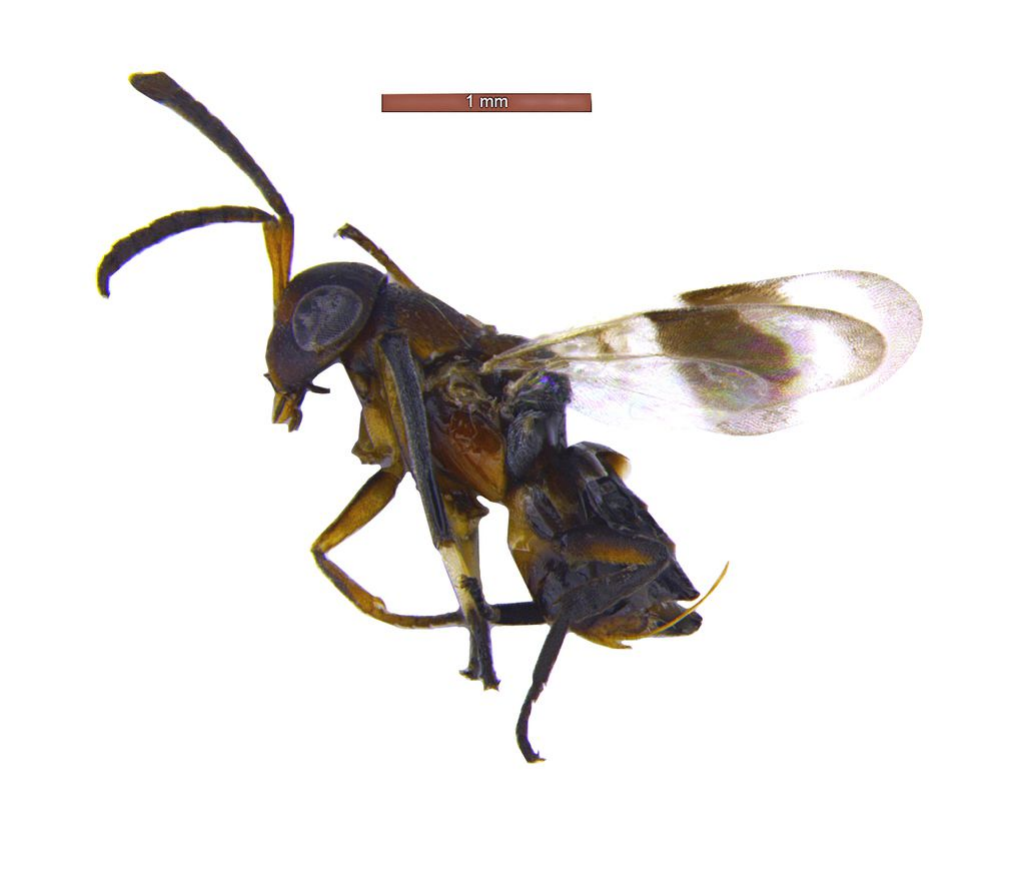
Habitus image Female

Body yellow to dark brown (Fig. 12); ocelli forming an angle of more than 90°; fore wing with three infuscate bands, middle one below marginal vein much larger, narrow faint vertical band below parastigma, and a wider band near apex; maxillary and labial palpus three segmented; scape, 6.30× as long as wide; pedicel, 1.5× as long as wide; F1, 2.16× as long a wide and slightly longer than F2−F3, much longer than F4−F6 individually; clava three segmented, obliquely trunctated; ovipositor 4.03× as long as mid tibial spur, curved and slightly exserted.

Female. (Fig. 12) Holotype. Length, 2.68 mm. Head dorsally dark brown to black including eyes, frontally yellowish brown, except the position of anterial tentorial pit, labrum and mandibles lower ¼ yellow, upper ¾ black; ocelli transparent. Antenna with radicle, scape yellow; pedicel and funicle ventrally pale brown, dorsally dork brown; clava black. Thorax: pronotum dark; mesoscutum basally dark, fading out to yellowish brown apically; axilla and basal ¾ of scutellum brownish yellow, apical ¼ black; mesopleuron anteriorly dark brown fading out to brownish yellow. Tegula basally black, apically transparent; fore wing with three infuscate bands, middle one below marginal vein much larger, narrow faint vertical band below parastigma, and a wider band near apex. Legs with fore coxa yellow, remainder of the leg dark brown dorsolaterally and ventrally yellowish brown; mid coxa brown, femur, tibia and tarsi dark brown to black except basitarsus, and tibial spur white with black apex; hind coxa brownish yellow except apex dark brown, femur basally and dorsolaterally dark brown, venterolaterally yellowish brown, remainder dark brown to black. Propodeum and gaster dark brown to black, except ventral base of gaster, apex of hpopygium and ovipositor yellowish brown.

**Figure 13.**
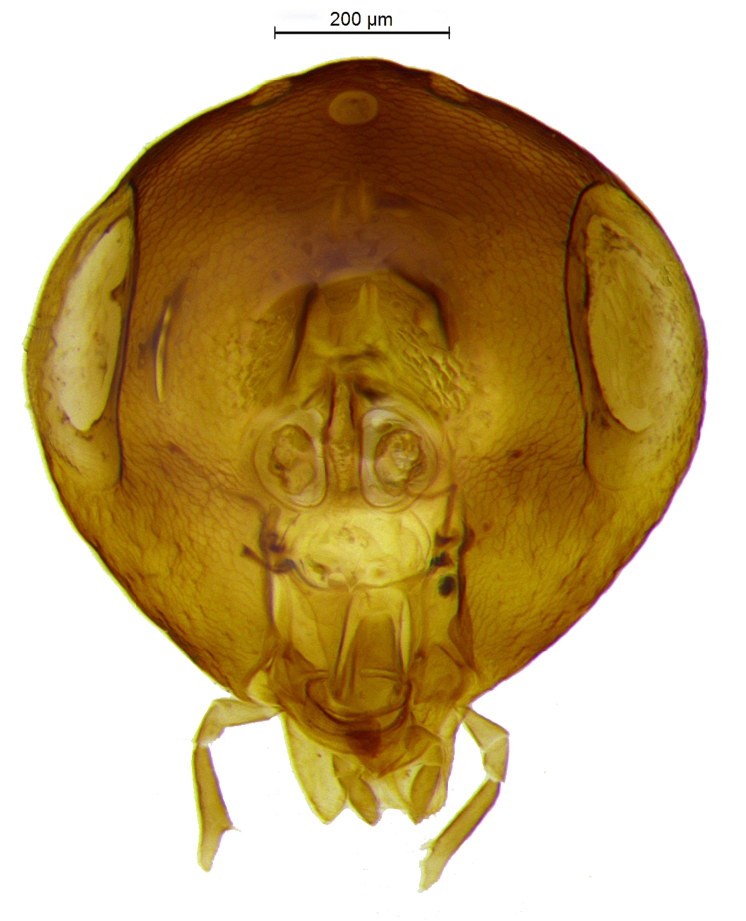
Head frontal view Female

**Figure 14.**
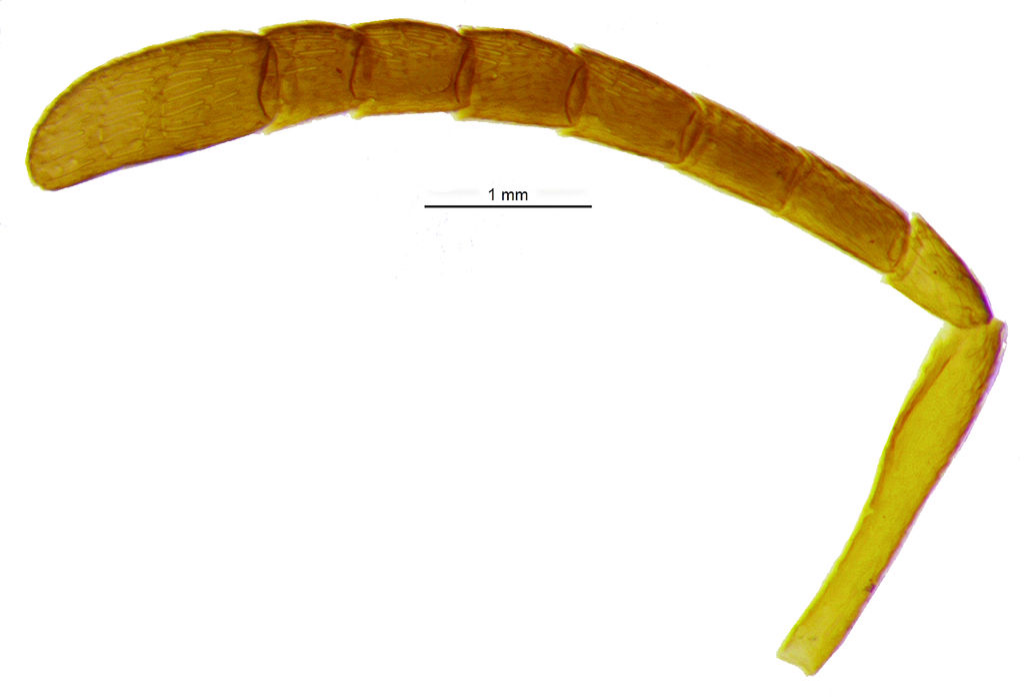
Antenna Female

Head (Fig. 13) in frontal view slightly longer than high (0.76:0.75), with silvery white setae, and frontovertex with imbricate, but cheek sharing coriarious punctate sculpture; ocelli forming an angle more than 90°; maxillary and labial palpi three segmented; inter antennal prominence raised; eye in frontal view 1.30× as long as wide. Antenna Fig. 14), with scape slightly flattened, 6.30× as long as wide; pedicel, 1.5× as long as wide; funicle with F1, 2.16× as long a wide and slightly longer than F2−F3, much longer than F4−F6 individually; clava three segmented, obliquely truncate, truncate area 0.36× of claval length; F1 and F2 without longitudinal sensillae; F3 to F4 and three claval segments respectively with at least 10, 11, 14, 16 14, 17 and 13 sensillae. *Measurements*: (card mounted 100×) eye height:width, 0.375:0.287; frontovertex width, 0.512; OOL 0.162; OCL 0.037; POL 0.162; AOL 0.087. (slide mounted 100×) head height:width, 0.76:0.75; scape length:width, 0.41:0.065; pedicel length:width, 0.12:0.08; funicle length:width, F1, 0.13:0.06; F2, 0.12:0.07; F3, 0.12:0.085; F4, 0.11:0.09; F5, 0.1:0.09; F6, 0.09:0.09; clava length:width, 0.27:0.11.

**Figure 15.**
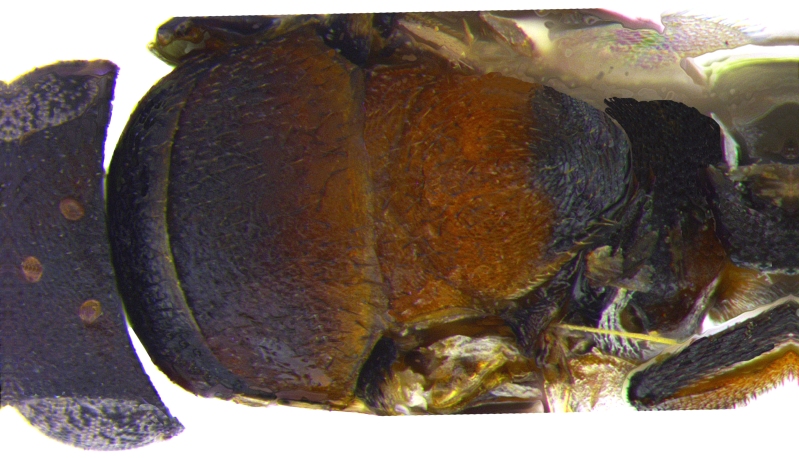
Dorsal view of nota Female

**Figure 16.**
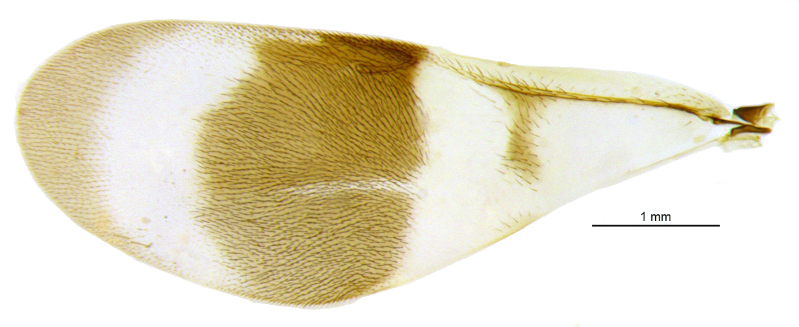
Fore wing Female

Mesosoma (Fig. 15) slightly longer than gaster (1.062:1), with imbricate sculpture; mesoscutum 2.16× as wide as long; scutellum as long as wide. Fore wing (Fig. 16), 2.68× as long as wide; costal cell 15.00× as long as wide; postmarginal vein 1.58× as long as stigmal vein; hind wing, 3.37× as long as wide. *Measurements*: (card mounted 100×) mesosoma length:width, 1.062:0.512; mesoscutum length:width, 0.375:0.812; scutellum length:width, 0.512:0.512. (slide mounted 100×) fore wing length:width, 1.96:0.73, costal cell length:width, 0.75:0.05; postmarginal vein length, 0.27; stigmal vein length, 0.17; hind wing length:width, 1.35:0.4; hind tibia length:width, 0.75:0.137, hind basitarsus length, 0.387; mid basitarsus length, 0.412; mid tibial spur length, 0.275; mid tibia length, 1.062.

**Figure 17.**
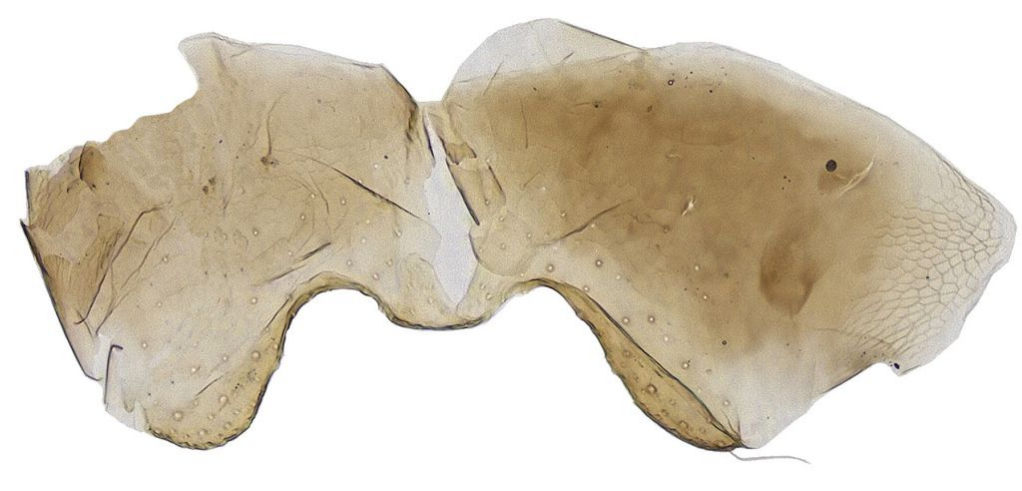
Hypopygium Female

**Figure 18.**
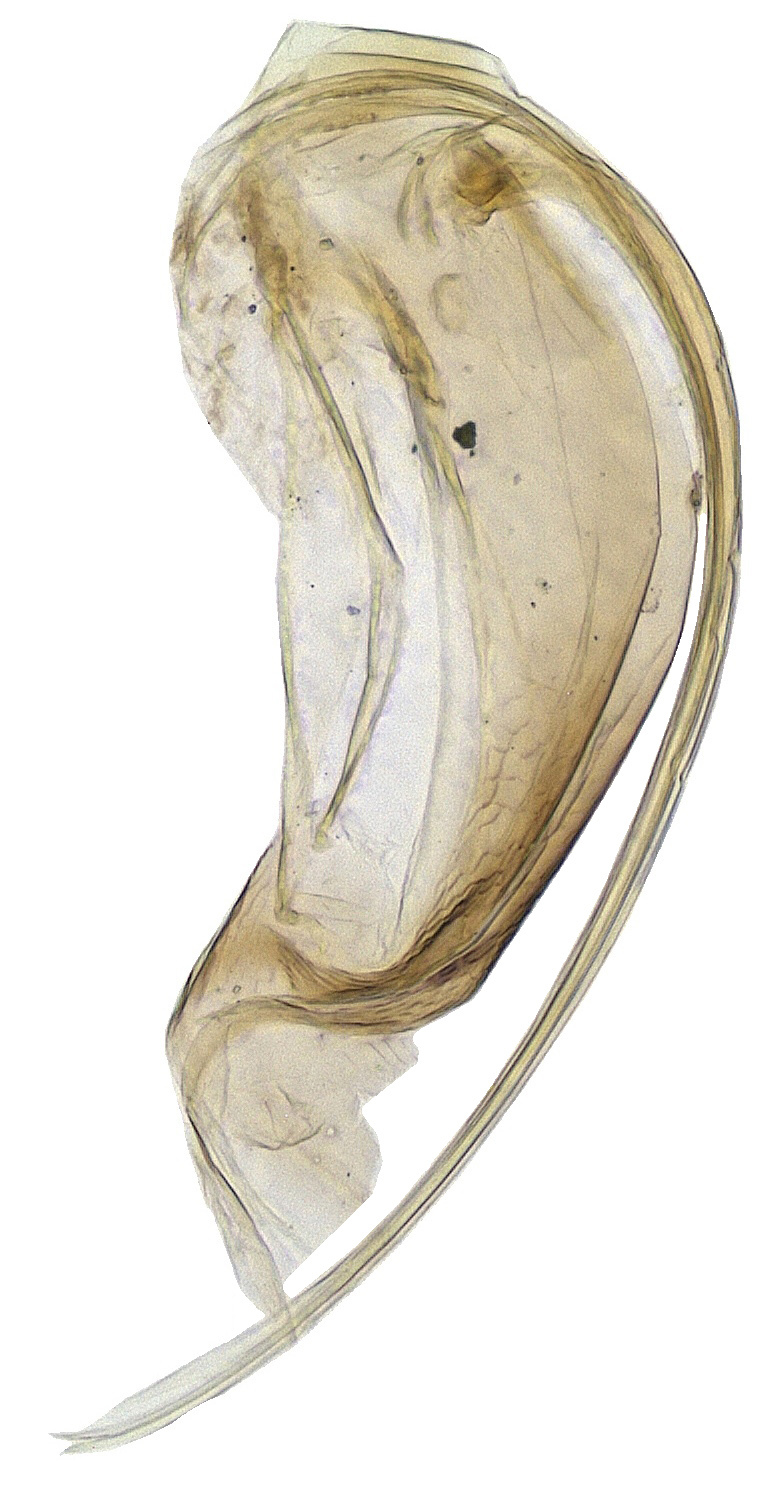
Ovipositor

Metasoma slightly shorter than thorax; gaster with imbricate sculpture; hypopygium (Fig. 17) nearly extending to apex of gaster; ovipositor (Fig. 18) 4.03× as long as mid tibial spur, curved and slightly exserted. *Measurements*: (card mounted 100×) metasoma length 1.00. slide mounted 100×) ovipositor length 1.11.

**Host.** Unknown.

**Male.** Unknown.

#### Etymology

The species is named after the state from where it was collected.

#### Notes

This species is closer to *P. orientalis* in having the mesosoma dorsally mostly brown or dirty yellow; longitudinal sensillae absent in F1 and F2; fore wing 2.68× as long as wide, but differs in having s cape 6.30× as long as wide; truncate area 0.36× of clava length; clava 2.45× as long as wide; postmarginal vein 1.58× as long as stigma vein; hind wing 3.37× as long as wide; F4 to F6 with 41 longitudinal sensillae (scape 5.60× as long as wide; truncate area 0.60× of clava length; clava 2× as long as wide; postmarginal vein 2.0× as long as stigma vein; hind wing 2.80× as long as wide; F4 to F6 with 15 longitudinal sensilla in *P. orientalis*).

## Identification Keys

### Key to Indian species of Prionomastix (Females)

**Table:** 

1	Fore wing hyaline, without any bands, but infuscate area around postmarginal and stigmal veins; scape at most 5.42× as long as wide; pedicel at most 0.73× as long as F1; fore wing at most 2.43× as long as broad	*P. tamilnadensis* **sp. nov.**
–	Fore wing infuscate, with 1 to 3 bands; scape at least 5.60× as long as wide; pedicel at least 0.92× as long as F1; fore wing at least 2.68× as long as broad	2
2	Scape 5.60× as long as wide; truncate area 0.60× of clava length; clava 2× as long as wide; postmarginal vein 2.0× as long as stigmal vein; hind wing 2.80× as long as wide; F4 to F6 with 15 longitudinal sensillae	*P. orientalis*
–	Scape 6.30× as long as wide; truncate area 0.36× of clava length; clava 2.45× as long as wide; postmarginal vein 1.58× as long as stigmal vein; hind wing 3.37× as long as wide; F4 to F6 with 41 longitudinal sensillae	*P. biharensis* **sp. nov.**

## Supplementary Material

XML Treatment for Prionomastix

XML Treatment for Prionomastix tamilnadensis

XML Treatment for Prionomastix biharensis
